# Functionalized scaffolds to enhance tissue regeneration

**DOI:** 10.1093/rb/rbu016

**Published:** 2015-01-11

**Authors:** Baolin Guo, Bo Lei, Peng Li, Peter X. Ma

**Affiliations:** ^1^Center for Biomedical Engineering and Regenerative Medicine, Frontier Institute of Science and Technology, Xi’an Jiaotong University, Xi’an 710049, China, ^2^Department of Biomedical Engineering, University of Michigan, ^3^Department of Biologic and Materials Sciences, University of Michigan, 1011, North University Avenue, Room 2209, ^4^Macromolecular Science and Engineering Center, University of Michigan, and ^5^Department of Materials Science and Engineering, University of Michigan, Ann Arbor, MI 48109, USA

**Keywords:** biomaterials, scaffolds, electrically conductive polymers, bioactive nanocomposites, bone tissue engineering, molecule-releasing scaffolds, antimicrobial coatings

## Abstract

Tissue engineering scaffolds play a vital role in regenerative medicine. It not only provides a temporary 3-dimensional support during tissue repair, but also regulates the cell behavior, such as cell adhesion, proliferation and differentiation. In this review, we summarize the development and trends of functional scaffolding biomaterials including electrically conducting hydrogels and nanocomposites of hydroxyapatite (HA) and bioactive glasses (BGs) with various biodegradable polymers. Furthermore, the progress on the fabrication of biomimetic nanofibrous scaffolds from conducting polymers and composites of HA and BG via electrospinning, deposition and thermally induced phase separation is discussed. Moreover, bioactive molecules and surface properties of scaffolds are very important during tissue repair. Bioactive molecule-releasing scaffolds and antimicrobial surface coatings for biomedical implants and scaffolds are also reviewed.

## Introduction

Tissue engineering provides a great promise in improving clinical therapy [1, 2]. By combining scaffolds with cells and bioactive molecules, tissue engineering seeks to generate an alternative functional tissue to repair injuries, in order to overcome the organ shortage in clinical treatment. The scaffolds play an important role during tissue regeneration [3, 4]. They provide a structurally relevant 3-dimensional (3D) environment that defines the shape of the tissue that needs regeneration [5, 6]. Scaffolds should have the key characteristics including: (i) highly interconnected porous structure which allows cell penetration and nutrient and waste transportation; (ii) biocompatibility and biodegradability, which are the basic requirement for the biomaterials used for scaffolds; (iii) suitable mechanical properties to meet the specific applications and (iv) appropriate surface modification and topography to support cell adhesion and growth. Various scaffolds have been developed for different tissue repair. Electrically conducting polymers have great potential application in tissue engineering, and has gained much attention recently [7]. A lot of works have shown that electrical stimulation can enhance the regeneration of muscle and nerve because they are sensitive to electrical stimulation. In this work, the development of conducting hydrogels and biomimetic conducting scaffolds for muscle and nerve regeneration was reviewed.

Different from the soft tissues such as muscle and nerve, human bone usually possesses the typical multi-component feature including organic collagen, elastic protein and inorganic apatite [8]. It is reasonable and helpful using biomimetic nanocomposites for bone tissue regeneration. Actually, biomimetic chemical compositions and structures in the bone-regenerative scaffolds have shown enhanced ability for cell biomineralization and osteogenesis [8]. Especially, bioactive ceramics including hydroxyapatite (HA) and silica-based bioactive glass (SBG), as the inorganic phases, have been widely used to reinforce the bioactivity and mechanical properties of scaffolds fabricated with organic polymers [9]. To mimic the nanofibrous structure and morphology of native tissue, composite nanofibrous scaffolds were also developed for enhanced bone regeneration [10]. Here, we summarized such different functional composite scaffolds for bone tissue engineering.

Bioactive molecules are also very important during tissue regeneration. The localized and temporally controlled delivery of bioactive molecules such as drugs and growth factors could enhance the clinical efficacy. It is beneficial for tissue engineering scaffolds to serve as both 3D substrate and bioactive molecules delivery depot to enhance cellular activity during tissue repair [11–13]. Therefore, the molecule-releasing scaffolds for tissue engineering are also discussed in the review.

When the scaffolds are transplanted into human body, infections on the scaffolds should be avoided. Despite advanced sterilization and aseptic techniques and extreme care taken during the implantation procedure, some scaffolds can still be infected by bacteria or fungi which can lead to severe infections or life threats [14–16]. Proper hygiene could only reduce the incidence of infections by two-thirds, but not eradicate it. The healthcare system has been longing for minimization of the risk of scaffolds-related infection [17]. There is an overwhelming demand of self-sterilizing biomaterials for regenerative medicine applications [18]. The approach and development of antimicrobial scaffolds are also summarized in this review.

## Conductive Hydrogel Scaffolds

**Figure 1.**
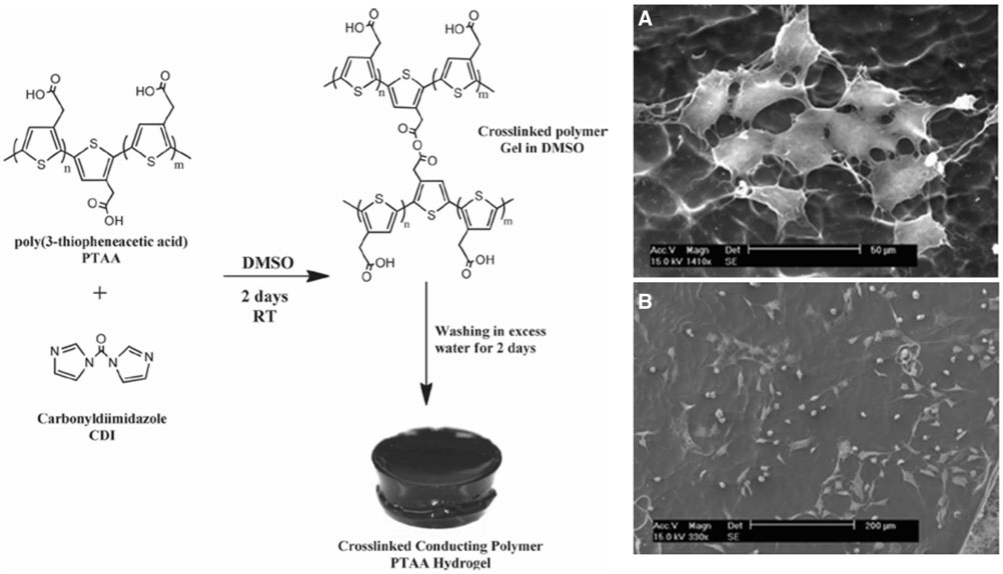
Schematic preparation of conductive hydrogel (left column). Representative SEM images of myoblast cells adhered to the conductive hydrogel substrates after 72 h incubation (right column) (A: high magnification, and B: low magnification) [19]. Copyright 2012. With the permission of Wiley.

Polymer hydrogels are 3D polymeric networks formed from hydrophilic monomers. Hydrogels are promising candidates as tissue engineering scaffolds [20, 21], due to their hydrated nature, good biocompatibility and their soft tissue-like mechanical properties. A single component conducting polymer hydrogel was fabricated by covalently cross-linking poly(3-thiopheneacetic acid) with 1, 1′-carbonyldiimidazole (Fig. 1) [19]. The mechanical properties of the conducting hydrogels were found to be comparable to that of muscle tissue. The hydrogels were electroactive and conductive at physiological conditions. Fibroblast and myoblast cells can adhere and proliferate on the hydrogel substrates. This work opens the way to developing conducting hydrogels as tissue engineering scaffolds based on conducting polymers. A biodegradable electroactive hydrogel based on aniline pentamer (AP) grafting gelatin (GA) is prepared [22]. The hydrophobic AP changes the porous structure of the natural GA hydrogel. The AP-g-GA showed reduced cytotoxicity than AP because of the introduction of the biocompatible GA component. Our group synthesized a series of degradable conducting hydrogels based on polycaprolactone (PCL), polylactide and aniline oligomers [23, 24]. For example, a simple and elegant synthesis route to obtain degradable and electrically conductive hydrogels (DECHs) was developed [25]. A series of DECHs based on acrylated poly (lactide)–poly(ethylene glycol)–poly(-lactide) macromer and aniline tetramer (AT) was synthesized. The conductivity varied between 1.05 × 10^−4^ and 4.69 × 10^−^^7^^ ^S/cm by tuning the AT content in the hydrogels. The swelling ratio was from 302% to 18.5% by changing the cross-linking density or the pH. These hydrogels which combine biodegradability from the polyester and conductivity from conducting polymers would lead to new application of conducting hydrogels, including drug delivery and tissue regeneration.

A facile route to DECHs composed of chitosan (CS) and AT was demonstrated [26]. A series of DECHs and their free-standing flexible films was prepared by a 1-pot reaction under mild conditions. The conductivity of the hydrogels changed from 2.94 × 10^–5^ to 2.97 × 10^–^^7^^ ^S/cm with AT content decreasing from 30% to 10%. The swelling ratio of the hydrogel is controlled by the AT content, the GA content and the pH values of the medium. To improve the non-solubility of CS in physiological solution and improve the conductivity of the hydrogels, carboxymethyl chitosan (CMCS) was used instead of CS due to its good solubility in aqueous solution, and AP was used to replace AT. Degradable conducting hydrogel composed of CMCS and AP was synthesized [27]. These hydrogels showed much higher swelling ratio and electrical conductivity compared with the CS–AT hydrogel. The C2C12 cell culture on the CMCS–AP hydrogels showed that these hydrogels are not cytotoxic.

The *in situ* forming ability of hydrogels gives the convenient administration in a minimally invasive way. A series of *in situ* forming biodegradable electroactive hydrogels was developed, which overcomes the poor processability of conducting polymers [28]. These hydrogels were synthesized by cross-linking gelatin-graft-polyaniline (PA) by genipin at physiological conditions. The gelation time, swelling ratio and degradation time of these injectable conductive hydrogels were tuned by the PA content and genipin content. The hydrogels released diclofenac sodium in a linear manner. The conductive hydrogels greatly enhanced the cell adhesion and proliferation of bone marrow mesenchymal stem cells and rat C2C12 myoblast cells, indicating that these materials have great potential for electrical sensitive tissue repair, such as bone, muscle and neural regeneration.

## Inorganic-Polymer Composite Scaffolds

### Biomimetic apatite-polymer nanocomposite scaffolds

For bone tissue repair and regeneration, traditional therapies include autogenous bone and allograft. Autogenous bone grafting has become a ‘gold standard’ for efficient bone regeneration. However, the limited donates and immunological diseases have restricted their applications for bone regeneration. Therefore, due to the controlled structure and properties, biomedical materials scaffolds have attracted much attention in recent years. Pure ceramic or polymer materials or scaffolds have been applied in bone tissue repair and regeneration, but these materials could not possess suitable mechanical properties and biocompatibility [29]. By mimicking the chemical composition and structure of native bones, it is reasonable to obtain new bone repair and regeneration biomaterials with suitable physicochemical properties and bioactivity. Here, we will summarize the recent development about biomimetic composition biomaterials scaffolds for bone tissue engineering.

Biomedical polymers (collagen, gelatin, CS, PCL and poly(lactic acid)) presented low modulus and poor bioactivity, and it is not satisfied to be used in bone tissue regeneration [30]. Bioactive ceramics, such as HA and bioactive glasses (BGs), possess good bone-bonding bioactivity but mechanical brittleness. Developing bioactive ceramic-polymer composite scaffolds has become the promising strategies for bone tissue repair and tissue engineering. Hydroxyapatite nanoparticle (HAN) is a very frequently used filler for preparing bioactive polymer composite scaffolds for bone tissue engineering. HAN-based collagen, gelatin, polysaccharide, PCL and silk fibroin (SF) composites scaffolds have been synthesized successfully [31–34]. Most of results showed that HAN incorporation can significantly increase the physicochemical properties, osteoblasts bioactivity and osteogenesis ability of polymer scaffolds. For example, after the reinforcement of HAN with 10%, poly (3-hydroxybutyrate) (PHB) scaffolds at a porosity of 77% showed a 2 times improvement of compressive strength and modulus, when compared with pure polymer [35].

### Biomimetic BG-polymer nanocomposite scaffolds

In addition to HAN, as another bioactive ceramic, bioactive glass nanoparticles (BGNs) were also used for fabricating polymer composite scaffolds for bone tissue engineering [36–38]. Different from HA, BG possesses a typical chemical composition of SiO_2_–CaO–P_2_O_5_ and amorphous structure. This amorphous structure makes BG good biodegradation and high bone-bonding bioactivity when implanting *in vivo*. BG-alginate (BG-ALA), BG-collagen (BG-COL) and BG-PHB composite scaffolds have been fabricated for bone regeneration applications [39–41].

Additionally, BG micro-nanoscale (BGMN) particles were also used to reinforce the strength and bioactivity of PCL which possesses high toughness but low stiffness and bioactivity. Lei et al. [42, 43] reported that the BGMN incorporation can significantly enhance the mechanical modulus (7 times improvement) and biomineralization bioactivity of PCL polymer. The *in vivo* studies also showed that BG-polymer composite scaffolds can significantly enhance the bone repair and regeneration compared with polymer scaffolds [44, 45].

### Biomimetic molecular-level bioactive silica-polymer hybrid scaffolds

**Figure 2.**
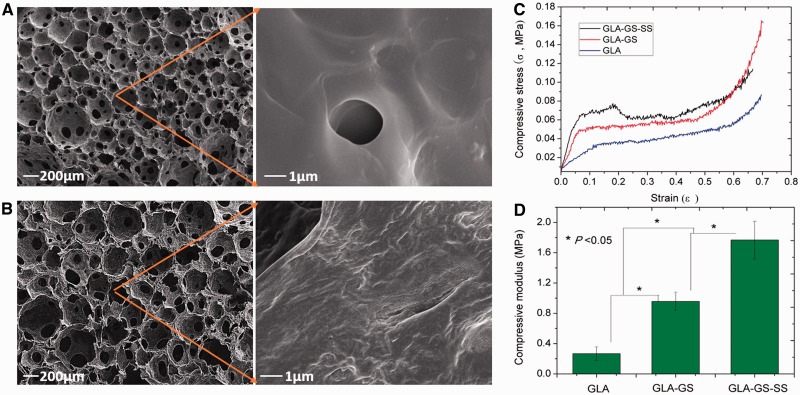
Typical gelatin–silica BG hybrid scaffolds prepared by direct foaming–freezing method. The molecular-level distributions in polymer matrix and significantly improved mechanical properties can be obtained after direct hybridization process. (A-B) Scaffolds morphology of pure gelatin (A) and gelatin-BG hybrids; (C-D) Stress-strain curves (C) and compressive strength (D) of samples (GLA: gelatin; GS: siloxane; SS: silica-based glass). Reproduced from Ref. [25] with permission from Elsevier.

Conventional BG particles were usually prepared by sintering at high temperature and they usually showed aggregative behavior in polymer matrix. It may benefit the properties of polymer composite scaffolds if the inorganic phase can present a molecular level distribution in polymer matrix. In recent years, due to the molecular-level feature, silica-based bioactive glass sol (SBGS) has been employed to fabricate polymer hybrid scaffolds. These hybrid scaffolds presented a significantly improved physicochemical properties and biocompatibility [46]. SBGS-based PCL, gelatin and CS scaffolds have been fabricated successfully [47–50]. For example, Lei and co-workers prepared the gelatin–silica hybrid scaffolds by direct foaming–freezing method (Fig. 2). These polymer hybrid scaffolds also exhibited significantly enhanced mechanical properties and osteoblasts bioactivity.

## Structural Biomimetic Scaffolds

### Nanofibrous electroactive scaffolds

**Figure 3.**
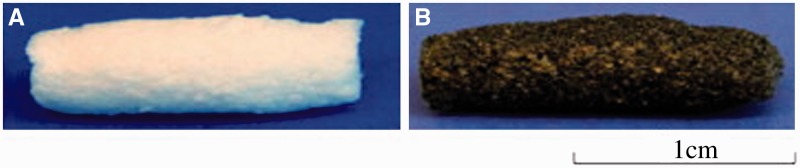
Photographs of the degradable tubular porous scaffolds from PCL (**A**) and degradable conductive porous scaffolds from blends of PCL/hyperbranched degradable conducting polymer (**B**) [53]. Copyright 2012. With the permission of Elsevier.

Various blends and composites were prepared from degradable polymers and conducting polymers. These materials overcome the disadvantages of conducting polymers such as non-degradability, low mechanical properties and non-solubility, and it is found that they are more suitable for tissue engineering application compared with conducting polymers alone [51]. These blends and composites are usually in the form of film or membrane, which do not show biologically relevant 3D structures that are important for tissue scaffolds [52]. Therefore, various techniques were used to create 3D structured conducting scaffolds for tissue regeneration application. For example, electroactive degradable tubular scaffolds based on blends of PCL and a hyperbranched degradable conducting copolymer were created by a modified solution-casting/salt-leaching method [53] (Fig. 3). The conductive scaffolds exhibited uniformly distributed interconnected pores on the cross-section and surface. The non-cytotoxicity of the conductive scaffolds was confirmed with water-soluble tetrazolium (WST) test with HaCaT keratinocyte cells. These degradable electroactive tubular scaffolds show great promise for neural tissue engineering. A conducting composite nerve conduit based on polypyrrole (PPY) and poly(d, l-lactic acid) (PLA) was prepared to support the differentiation of rat PC12 cells and to enhance nerve regeneration *in vivo* [54]. A significant increase in the percentage of neurite-bearing cells and the median neurite length was found when the PC12 cells on conduits were stimulated with 100 mV for 2 h. Furthermore, the PPY/PLA nerve conduit performed similarly to the autologous graft when it was used to repair a rat sciatic nerve defect, indicating that the PPY/PLA conducting conduit has great potential for neural tissue engineering.

Many extracellular matrices (ECMs) possess a fibrous structure with diameters in the range between nanometer and sub-micrometer. For instance, the most abundant ECM protein collagen shows a nanofibrous structure with fiber size between 50 and 500 nm. To better mimic the micro-structure of ECM, tissue engineering scaffolds are fabricated with nanofibrous features. The nanofibrous scaffolds provide interconnecting pores and large surface areas, which are beneficial for cell attachment, cell in-growth and the exchange of nutrients and wastes. Up to date, 3 major fabrication methods for nanofibrous scaffolds are most widely used: self-assembly, electrospinning and phase separation [55–57]. Among these technologies, electrospinning is one of the most versatile and robust techniques for producing nanofibers [58–62]. Electrospinning conducting polymers alone to uniform nanofibers are quite difficult because of the polymer backbone rigidity, and their low solubility in common organic solvent. To overcome these problems, conducting polymers are usually mixed with various electrospinnable polymers to increase electrospinning processability. The blends based on poly(lactic acid-co-glycolic acid) (PLGA) and poly(3-hexylthiophene) (PHT) were electrospun into 2D random and 3D axially aligned nanofibers [63]. The pore size of aligned PLGA/PHT nanofibers was significantly lower than the random ones, and Young’s modulus of random scaffold was significantly higher than the aligned ones. Aligned nanofibers exhibited lesser degradation rate and much higher electrical conductivity than random ones. Schwann cell culturing results on the aligned PLGA–PHT nanofibers showed a great influence on the adhesion and proliferation of the cells, indicating the potentials as scaffold for neural tissue engineering.

Conductive nanofibrous scaffolds composed of PPY/PCL/gelatin (PPY/PCL/PG) were electrospun by incorporating different concentrations of PPY to PCL/PG. With increasing the concentration of PPY (0–30%) in the blends, the average fiber diameters reduced, and the tensile modulus of the scaffolds increased [64]. Nanofibers containing 15% PPY (PPG15) in the composites exhibited the most balanced properties including conductivity, mechanical properties and biodegradability. The cell proliferation assay demonstrated that the PPG15 scaffold promoted cell attachment, proliferation and the expression of cardiac-specific proteins higher than PPG30, which is promising substrates suitable for the repair of cardiac defects.

Scaffold should provide appropriate guidance cues for particular cell types for modulation of cell behavior. The highly aligned and electroactive nanofibers can simultaneously provide topographical and electrical cues to regulate cell behavior. Well-ordered electrospun nanofibers based on PA and PCL were fabricated [65]. Introduction of PA into PCL fibers greatly increased the electrical conductivity to 63.6 ± 6.6 mS/cm when 3 wt% PA was added (PCL/PA3). C2C12 myoblasts were cultured on random PCL (RPCL), aligned PCL (APCL), RPCL/PA3 (RPCL/PANi) and APCL/PA3 (APCL/PA) nanofibers to study the effects of topographical and electrical cues of the nanofibers. The aligned scaffolds (APCL and APCL/PA) could guide C2C12 cell orientation and promote myotube formation compared with RPCL nanofiber. Furthermore, conductive APCL/PA scaffolds enhanced myotube maturation compared with insulating APCL or RPCL/PA. These data indicated that the combined effect of both topographical and electrical cues was more effective than an individual cue.

**Figure 4.**
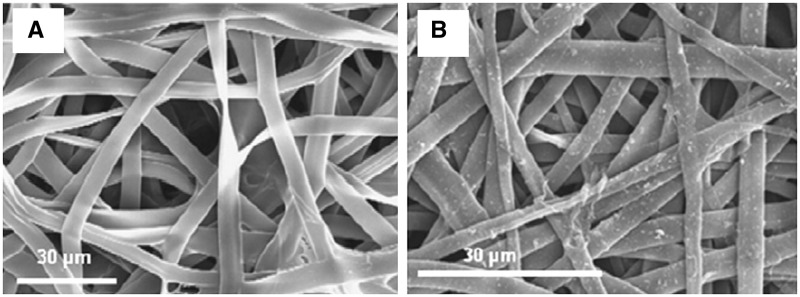
SEM images of the nanofibers. (**A**) Superficial aspect of non-coated SF mesh and (**B**) SF-PPY-coated mesh [66]. Copyright 2012. With the permission of Elsevier.

Another method for fabricating conducting scaffolds is deposition of conducting polymers on the surface of the electrospun nanofibers [67]. For example, electrospun nanofibers of SF were coated with PPY by chemical polymerization, and they found that mechanical resistance of the scaffolds was improved by PPY coating [66] (Fig. 4). In addition, coated meshes showed a high electroactivity allowing anion storage and delivery. The PPY-coated meshes supported the adhesion and proliferation of adult human mesenchymal stem cells or human fibroblasts. Poly-96l/4d-lactide (PLA) nonwoven scaffolds were coated by chondroitin sulfate-doped PPY via *in situ* chemical oxidative polymerization as novel osteogenic scaffolds [68]. PLA–PPY scaffolds significantly enhanced the proliferation of human adipose stem cells (hASCs) compared with the PLA scaffolds. Moreover, the alkaline phosphatase (ALP) activity of the hASCs was higher for PLA–PPY scaffolds. These results highlight the promise of PPY-coated PLA scaffolds for bone regeneration.

The bioactive molecules such as growth factor play an important role in tissue regeneration. Scaffolds with the combination of multiple cues such as submicrometer-scale features, electrical conductivity and neurotrophic activity for neural tissue regeneration were fabricated by electrospinning based on poly(lactic acid-co-glycolic acid) (PLGA) and electrically conducting PPY bearing carboxylic groups [69]. The nerve growth factor (NGF) was then chemically coupled onto the surface of the fibers. These NGF-immobilized conductive PPY-coated PLGA (NGF–PPY/PLGA) fibers enhanced PC12 neurite formation and neurite outgrowth. Electrical stimulation on NGF–PPY/PLGA scaffolds further enhanced neurite development and neurite length, compared with unstimulated cells on the NGF-immobilized fibers. Hence, submicrometer-scale fibrous scaffolds that incorporate neurotrophic and electroconducting activities may serve as promising neural tissue engineering scaffolds such as nerve guidance conduits.

### Biomimetic nanofibrous composite scaffolds

Although nanocomposite scaffolds have shown many positive results for bone tissue engineering, conventional scaffolds usually presented limited osteogenic bioactivity because they can only mimic the chemical composition, but not the nanostructure and morphology of bone extracellular matrix (ECM) (nanofibrous structure). It is necessary to develop composite nanofibrous scaffolds for improving bone repair and regeneration [55].

To prepare nanofibrous polymer scaffolds, electrostatic spinning and phase separation methods were usually employed. To obtain composite structure, inorganic nanoparticles were usually incorporated into polymer solution before forming nanofibers. Similar with conventional nanocomposite scaffolds, HAN and BGN were usually chose as bioactive inorganic phase. By electrospun method, HAN-chitosan (HAN-CTS), HAN-SF and HAN-PCL nanofibrous scaffolds have been prepared successfully [70–72]. For example, HA reinforced CS nanofibrous scaffolds showed significantly high ALP activity and osteonectin mRNA expression, when compared with pure CS nanofibrous scaffolds.

**Figure 5.**
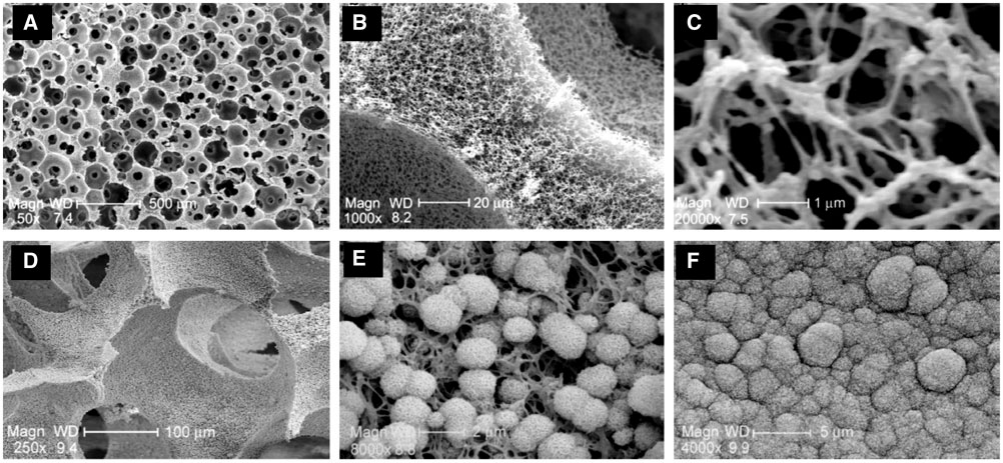
Biomimetic gelatin and gelatin–apatite nanofibrous scaffolds fabricated by TIPS and biomineralization methods. (**A–C**) Morphology and microstructure of nanofibrous scaffolds; (**D–F**) scaffolds after biomineralization for 7 days (D and E) and 21 days (F). Reproduced from Ref. [31] with permission from Elsevier.

Electrospun-derived nanofibrous scaffolds showed limited pore size ranging from several micrometers to 100 µm which is unfavorable for cell infiltration and growth. To address this problem, thermal induced phase separation (TIPS) technique was developed to fabricate macroporous nanofibrous scaffolds. Ma and co-workers have done much pioneering work about TIPS nanofibrous scaffolds for bone tissue engineering [73]. For example, Ma et al. fabricated the gelatin and gelatin–apatite composite nanofibrous scaffolds by TIPS method and apatite layer coating after biomineralization in simulated body fluid (SBF), as show in Fig. 5 [74]. The coated gelatin composite nanofibrous scaffolds showed significantly high mechanical strength and enhanced osteogenic genes expressions. The improved biocompatibility of nanofibrous composite scaffolds for bone tissue regeneration could be attributed to their biomimetic and biomineralized bone extracellular environment.

In addition to the apatite-based polymer nanofibrous scaffolds, silicate-based bioactive glass reinforced polymer nanofibrous scaffolds have also attracted much attention in recent years. BGNs incorporated gelatin and poly(ε-PCL) nanofibrous scaffolds have been fabricated by electrospun techniques [75, 76]. BGN addition significantly increased the mechanical properties and biomineralization ability of polymer nanofibrous scaffolds. Additionally, to improve BGN distribution in polymer matrix, silica-based bioactive sol-based polymer nanofibrous scaffolds were first developed by Lei et al. [77] using TIPS method. In this study, bioactive silica could be hybridized with polymer at a molecular level without any visible phase separation. Significantly high mechanical strength and biominerialization activity were observed from hybrid gelatin–silica nanofibrous scaffolds. Compared with HA, BG-based composite nanofibrous scaffolds did not achieve wide and deep study, which may be because it is difficult to prepare mono-dispersing nanoparticles.

## Biomolecule-Releasing Scaffolds for Tissue Engineering

The biomimetic composite scaffolds described above could provide a physical support for cell proliferation and differentiation. However, only physical support is not enough for ideal bone tissue engineering, biomolecule-releasing ability is preferable for improved bone regeneration efficiency. Here, bone growth factor and anti-infection drugs were usually loaded in composite scaffolds for functional bone tissue engineering applications [78, 79]. The addition of bioactive molecules into composites scaffolds can greatly accelerate the bone tissue regeneration ability.

**Figure 6.**
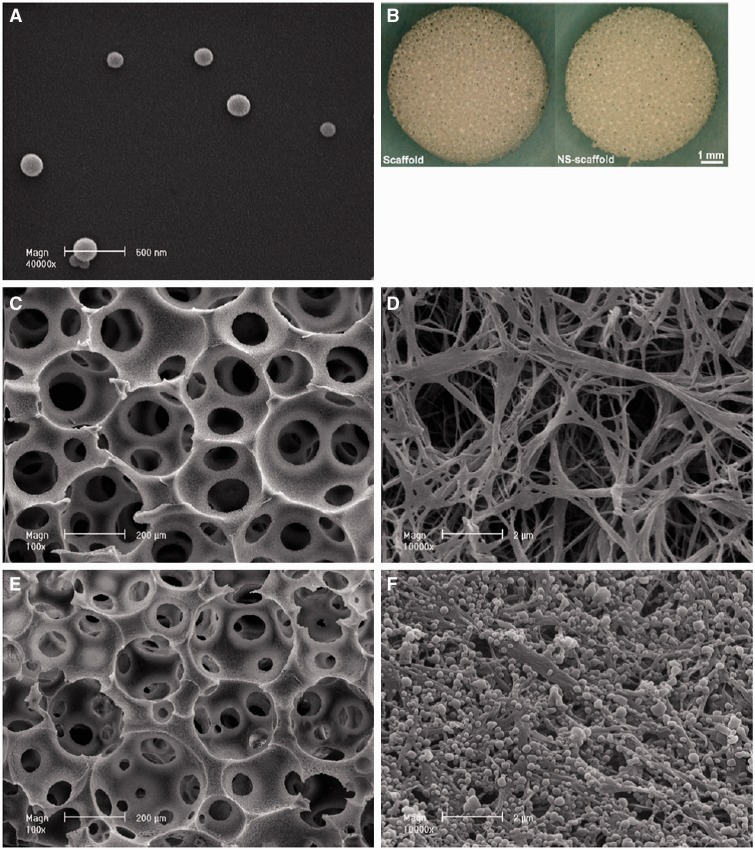
Functionalized PLA nano-fibrous scaffolds incorporating recombinant human bone morphogenetic protein-7 (rhBMP-7) nanospheres. (**A**, **B**) rhBMP-7-loaded nanosphere and scaffolds; (**C–F**) SEM images of nano-fibrous scaffolds before (C and D) and after nanosphere incorporation (E and F). Reproduced from Ref. [31] with permission from Elsevier.

Bone cells can be only responsive to certain concentration of growth factors, so the controlled release in 3D scaffolds seems to be very important for efficient bone regeneration [37]. Liu et al. [80] fabricated a bone morphogenetic protein (BMP-2)-loaded gelatin–HA composite scaffolds for segmental bone regeneration. The results showed that biomolecule-releasing scaffolds can significantly enhance bone marrow stem cell osteogenic differentiation and repair the segmental bone defect completely in 12 weeks. In addition, the growth factors (BMP) loaded in composite scaffolds could present a sustained release, which could accelerate the bone response process. Other growth factors such as vascular endothelial growth factor, transforming growth factor β and growth/differentiation factor 5 were also loaded in scaffolds for improved tissue engineering applications [81, 82]. Ma and co-workers fabricated a 3D nanofibrous scaffolds with the capability of controlled releasing BMP-7 [83], as shown in Fig. 6. The growth factors were first encapsulated into the polymer microspheres and then immobilized onto nanofibrous scaffolds. These functional scaffolds with controlled BMP-7 releasing ability showed significantly high ectopic bone formation ability compared with the scaffolds by passive adsorption of BMP-7.

## Antimicrobial Modification for Scaffolds

### Antimicrobial release scaffolds

**Figure 7.**
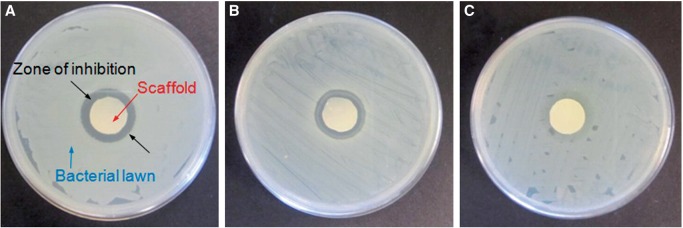
Antimicrobial properties of PLA nanofibrous scaffolds treated with Silvadur ET containing 31.25 μg/ml silver against *Escherichia coli* (**A**), *Staphylococcus aureus* (**B**) and silver-resistant *E. coli* (**C**) bacteria as evaluated by the AATCC 147 test [92]. Copyright 2014. With the permission of Elsevier.

During the implantation of scaffolds, even with the help of hygienic technique during the operation, opportunistic microbes still manage to be introduced to the implant sites [84]. Host defenses are often not capable of preventing further colonization if bacterial adhesion occurs before tissue integration at the implant [15, 85]. Antimicrobial modification of scaffolds based on drug-release strategy has been very popular in recent years, and these may be designed to release antimicrobial molecules to inhibit microbial colonization in the surrounding environments. Most commonly, the scaffolds are loaded with antimicrobials such as antibiotics [86, 87], quaternary ammonium compounds [88], heavy metal compounds (e.g. silver, tributyltin and mercury) [89, 90] and halogens (e.g. iodine) [91], which are then slowly released into the environment to kill microbes around. Figure 7 shows PLA nanofibrous scaffolds releasing silver ions which inhibit the bacteria growth [92].

These leaching systems are useful to prevent the scaffold implantation-related infections. However there are still some disadvantages. Normally these technologies require much larger quantities of the antimicrobial reagent than actually needed since they are gradually released, and they also pose great health hazards and contamination to the surrounding environment. Since the antimicrobial reagent is free to release from the surface, it shall be eventually exhausted and so has limited useful lifetime. Moreover, the continuous and mostly unnecessary release of biocides promotes development of microbial resistance, which has been recognized as one of the major problems in modern public health [93, 94].

### Contact-active antimicrobial coatings for scaffolds

Non-leaching surfaces that kill microbes effectively by contact represent a new approach to antimicrobial modification for biomaterials [95]. Contact-active antimicrobial surface coatings can be fabricated by permanent immobilization of biocides by various techniques such as depositing [96], layer by layer [97] or surface grafting [16, 98–100].

Titanium dioxide has been successfully deposited onto polydimethylsiloxane surface by liquid phase deposition and the adhesion of both Gram-negative and Gram-positive bacteria on the modified surfaces has been reduced [96]. Layer-by-layer technique is also popular in building up an antimicrobial surface. An antimicrobial peptide (AMP) Chromogranin A was embedded on poly(methylmethacrylate) surface, the growth of *Candida albicans* has been inhibited by 65% and the proliferation was completely stopped [97]. The other approach to produce contact-active antimicrobial surfaces is binding antimicrobial reagents to the surface through covalent interactions. Antimicrobial coatings developed from covalent bonding are permanent compared with biocide-release strategies.

**Figure 8.**
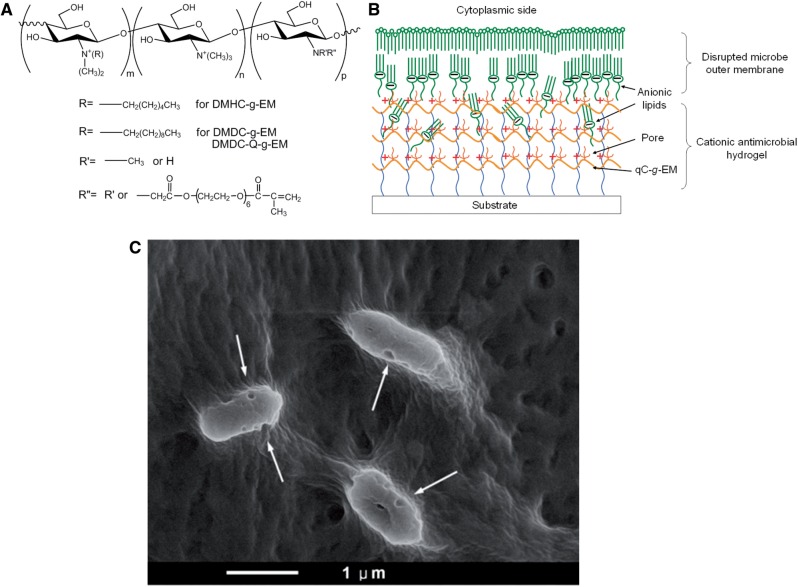
Nanoporous antimicroibial hydrogel coating fabricated by polysaccharides: (**A**) synthesis of quaternized CS functionalized with acrylate PEG side-chains; (**B**) formation of nanoporous hydrogel coating which is capable to kill microbes; (**C**) the cell wall of the Gram-negative bacteria *Pseudomonas aeruginosa* was disrupted by the nanoporous hydrogel. Reproduced from Ref. [105] with permission from Nature Publishing Group.

Surface modification by immobilization of AMPs is a promising method to prevent infections. Peptide LL-37 was grafted on titanium surface with a polyethylene glycol spacer by Gabriel et al. [101], which resulted in a surface peptide layer capable of killing bacteria on contact. Zhou and coworkers developed broad-spectrum antimicrobial surface coating by the immobilization of epsilon-poly-l-lysine hydrogel onto biomedical devices [102]. Besides antimicrobial activity, several recent AMP-immobilized surface coatings also show excellent anti-biofilm activities [103, 104]. Recently, Li et al. have developed novel broad-spectrum antimicrobial coating materials based on natural polysaccharides [105, 106]. Firstly, a group of antimicrobial materials were synthesized by quaternization and alkylation of CS. An argon plasma-ultraviolet (UV)-induced coating method for hydrogel surface immobilization was developed, which can be applied on diverse biomedical surfaces. A novel mechanism of these hydrogels based on ‘anion sponge’ concept was proposed and proven (Fig. 8). The optimized coating formulation and conditions show excellent antimicrobial potency. The *in vitro* and *in vivo* studies suggest this antimicrobial coating is biocompatible with mammalian cells.

## Conclusions

Tissue engineering is an emerging interdisciplinary field with the purpose of repairing or enhancing tissue function and it will greatly improve the quality of human life. Scaffolds during tissue repair should be analogous to native ECM both in chemical composition and physical structure. Conducting polymers substrates provide the potential of electrical stimulation on the scaffolds, and greatly enhance the organ regeneration of electroactive tissue such as nerve and muscle. Electrical conducting scaffolds have been fabricated via solution casting/particle leaching method, electrospinning of conducting polymer blends with other polymers and conducting polymer deposition on template scaffolds. Biologically active molecules can also be immobilized on conducting scaffolds, which renders the scaffolds multifunctional clues for tissue engineering. Furthermore, the interactions between the neural and cardiac cells and conducting scaffolds have also been studied. Therefore, fabrication and tissue engineering applications of conducting scaffolds will have great impact on tissue engineering and other bio-related area.

For bone regeneration, many studies have focused on developing inorganic bioceramic nanoparticles reinforced polymer scaffolds. The composite scaffolds took advantages of biomimetic structure, osteoconductivity and mechanical strength of inorganic ceramics. Considered on the nanofibrous morphology of native collagen, further studies aimed at developing composite nanofibrous scaffolds. Due to the biomimetic feature, nanofibrous composite scaffolds may continue to be the foundation for bone tissue engineering. To further improve tissue regeneration ability, growth factor or protein could be loaded in biomimetic scaffolds. For biomaterials scaffolds, as a structure support, the mechanical strength of nanofibrous scaffolds should be further improved to match new tissue formation. For biomolecules delivery, the controlled releasing behavior and loading efficiency should be further enhanced. To achieve these points, bioactive and molecular-level inorganic–organic nanofibrous hybrid scaffolds may play an important role on tissue engineering. In addition, more *in vivo* studies should be done to illustrate the potential and promise of as-prepared scaffolds.

As the fast growing of tissue engineering, more and more scaffolds were adopted in medical treatments. Despite advanced sterilization and aseptic techniques and procedures, infections associated with medical implants/devices have not been eradicated, particularly in long-term implants. Antimicrobial coating is a promising approach to lower the incidence of infections. Here, we summarized two different strategies of the design and fabrication of antimicrobial scaffolds, which have a great potential in regenerative medicine applications. These antimicrobial coatings would lower down the infections incidence and beneficial to human health.
